# Performance of the Self‐Controlled Case Series With Active Comparators for Drug Safety Signal Detection Using the Clinical Practice Research Datalink (CPRD)

**DOI:** 10.1002/pds.70243

**Published:** 2025-12-12

**Authors:** Astrid Coste, Angel Y. S. Wong, Francois Haguinet, Andrew Bate, Ian J. Douglas

**Affiliations:** ^1^ LSHTM, Department of Non‐Communicable Disease Epidemiology London UK; ^2^ GSK Global Safety Wavre Belgium

**Keywords:** Clinical Practice Research Datalink, pharmacoepidemiology, pharmacovigilance, real‐world data, self‐controlled case series, signal detection

## Abstract

**Background:**

There is little evidence about signal detection using UK primary care electronic health records (EHRs). The self controlled case series (SCCS) is one of the most promising methods for drug safety signal detection using real world data, and incorporating active comparators could potentially improve its performance by addressing confounding by indication.

**Objectives:**

This study aims to evaluate the performance of the SCCS with and without active comparators for signal detection using the UK Clinical Practice Research Datalink (CPRD) Aurum.

**Methods:**

We applied the SCCS to macrolide and fluoroquinolone antibiotics, using amoxicillin and cefalexin as active comparators. In total seven drugs, and 30 outcomes from all organ classes were selected. We developed a reference set of 104 positive controls and 58 negative controls, using a taxonomy framework to ensure the selected drug outcome pairs are theoretically well suited to the SCCS design. Two‐year observation periods with a 30‐day risk window after each dispensing were used. Diagnostic performance was measured using sensitivity and specificity with respect to the product labels.

**Results:**

The sensitivity and specificity of the SCCS without active comparator in the 2017/2018 observation period were 0.57 and 0.77 when limited to pairs with satisfactory power. Specificity increased up to 0.89 with active comparators, however sensitivity decreased to 0.18. Five drug‐outcome pairs were signals of disproportionality before they were present on labels.

**Conclusions:**

Using a carefully designed reference set of drug‐outcome pairs well suited to the study design, the SCCS performed moderately well for signal detection in CPRD. Whilst active comparators effectively reduced confounding by indication, they also reduced the number of correctly identified positive controls, due to a reduction in power. We showed some evidence that SCCS is able to highlight SDRs before they were present on labels.

**Summary**

- The self‐controlled case series (SCCS) is a promising method for drug safety signal detection using real‐world data but evidence is lacking in the United Kingdom Clinical Practice Research Datalink (CPRD).
- We explored SCCS performance in CPRD with and without active comparators using a tailored reference set based on macrolide and fluoroquinolone antibiotics drug labels.
- The sensitivity and specificity of SCCS without active comparator were 0.56 and 0.77 when limited to pairs with satisfactory power.
- Five drug‐outcome pairs were highlighted by the method before they were added to drug labels, showing capability of timely signal detection.
- SCCS performed moderately well for signal detection in CPRD. Active comparators effectively reduced confounding by indication, but they also reduced the number of correctly identified positive controls.

## 1 Introduction

UK electronic health records (EHRs) databases have been long used for pharmacoepidemiologic studies and the evidence generated from them is well established as a trusted source for public health decision making [1]. Whilst real world data (RWD) sources, including UK EHRs, have traditionally been used to confirm alerts arising from spontaneous reports (SRs), they can also play a role in signal detection, complementing spontaneous reporting systems. However, their relative value compared to SRs for signal detection is not well established [2]. In particular, the Clinical Practice Research Datalink (CPRD) is one of the largest and most utilised datasets in Europe for real‐world evidence studies.

To determine the value of RWD for signal detection, there is a need to evaluate the performance of traditional epidemiological methods at finding alerts in a hypothesis‐free context that is, irrespective of whether there is a suspected association. In practice, this is limited by the requirements of study designs, which can better accommodate certain types of drugs and outcomes. Previous RWD signal detection initiatives that explored method performance did not consider the fact that epidemiological methods are differentially valid depending on the nature of the drug and outcome [3].

Among investigated study designs for signal detection in RWD, the self‐controlled case series (SCCS) achieved consistently higher performances than other methods [4, 5, 6, 7, 8]. Self‐controlled designs present the advantage of inherently adjusting for time‐independent confounders. However, they are subject to confounding by indication since this type of confounding is time‐dependent [9]. Active comparators enable the handling of time‐varying confounding by indication and have been recently implemented in self‐controlled designs in hypothesis testing studies using Danish registry data [9] as well as in CPRD Aurum and Gold [10]. However, active comparators with SCCS have not been evaluated for signal detection so their impact on the method's performance remains unknown.

CPRD Gold has been used for near‐real time vaccine surveillance, and has been noted as a potential resource for signal detection [11, 12]. Other UK EHRs databases such as The Health Improvement Network (THIN) have been investigated for signal detection in a small number of studies [13, 14]. One study [13] used the SCCS to study recently approved products and investigated three drugs and nine outcomes in total, and found that SCCS highlighted all acute outcomes but missed some of the slower‐onset ones.

This is the first study to evaluate the performance of the SCCS with and without active comparators for signal detection in CPRD Aurum. We applied a pre‐determined reference set of labelled outcomes, developed using a taxonomy framework to ensure the selected drug outcome pairs are theoretically well suited to the SCCS design. Using a drug‐based approach, antibiotics were chosen for this study as they are prescribed in short courses in the United Kingdom, which are well suited to the SCCS design. Antibiotics are medications used to treat bacterial infections and have a well‐established safety profile since they have been on the UK market for several decades, which makes them well suited for performance assessment.

## 2 Methods

### 2.1 Data Source

CPRD Aurum consists of EHRs from patients registered at 1489 general practitioner (GP) practices in the United Kingdom. All patients registered with these practices are included in the database unless they have opted out [15]. With records for 39 million total and 13 million active patients as of March 2021, representing about 20% of the UK population [16], CPRD is considered a relatively large database for most epidemiological questions. CPRD patients are broadly representative of the UK population in terms of age and sex [15]. We only used de‐identified patient‐level data; therefore individual informed consent was not required.

Strengths of CPRD include the data quality, representativeness and length of follow‐up [15]. Notably, demographics (age, sex, death date, region), some health measurements and lifestyle factors (body mass index, smoking status), symptoms and diagnoses, prescriptions, referrals, vaccines, lab tests and results are recorded in CPRD. Some of its limitations include the lack of standardised diagnosis definitions in the SNOMED coding system and data that are not captured, such as over‐the‐counter medications, prescriptions in secondary care or adherence to treatment.

While CPRD is often used in pharmacovigilance for proactive monitoring of risks and benefits of drugs and vaccines [17], it has, to our knowledge, not been used in any of the large signal detection initiatives, in particular those looking at methods' performance.

### 2.2 Study Design

**FIGURE 1:**
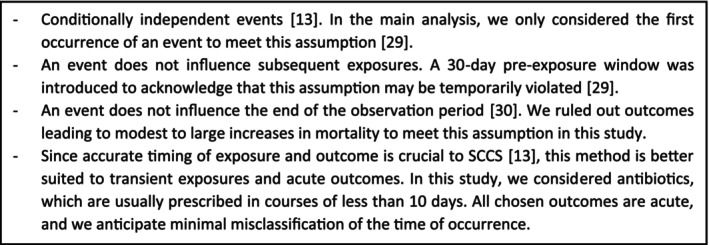
Main assumptions of the SCCS method.

The SCCS is a case‐only design comparing the event rate during exposed and unexposed time within the same individual [18]. Since all comparisons are made within person, between‐person confounding is inherently addressed. The main assumptions of the method are listed in Figure 1.

One limitation with this method is that time‐varying confounding is not handled by design. Active comparators have been recently implemented with SCCS [9] to reduce time‐varying confounding by indication. Therefore, we chose to investigate their impact on the signal detection performance of SCCS.

### 2.3 Reference Set

We built a reference set specifically for this study using a taxonomy framework to ensure the selected drug outcome pairs are well suited to the SCCS design. The reference set contains 104 positive controls (drug‐outcome pairs) and 58 negative controls in total, based on 30 outcomes from all organ classes. The full process has been detailed in a previous paper [19].

Positive control outcomes were selected among adverse drug reactions (ADRs) present on UK labels (taken from www.medicines.org.uk/emc), on an individual drug basis. The choice of negative controls is particularly challenging given the evolving nature of drug safety knowledge, both in terms of what causes or does not cause health outcomes. In this study, negative control outcomes were selected using the same list of positive control outcomes, since they are theoretically well suited to the SCCS design. We chose negative control outcomes specifically for each drug, requiring that the outcome is not labelled as an expected ADR for any of the drugs in the same drug class. For example, amnesia is a positive control (listed ADR) for moxifloxacin but is not on any of the macrolides' labels and as such is a negative control for all macrolides. Two negative controls were added on top of this list, which have been considered in previous signal detection studies: hip fracture and upper gastrointestinal bleeding [7, 20]. Only clinically important outcomes meeting the SCCS requirements and appropriately captured in CPRD Aurum were included in the reference set [19].

### 2.4 Study Population and Design Choices

We considered two different observation periods—each 2 years in duration: 1 January 1997 to 31 December 1998 and 1 January 2017 to 31 December 2018. The first observation period was chosen as some outcomes of interest were not listed as expected ADRs on labels at the time; therefore minimising bias related to clinicians being aware of the associations. The 2017–2018 period was selected based on data availability at the time of study inception and prior to the Covid‐19 pandemic to avoid potential changes in access to care or prescription patterns. A 2‐year study period was intended to mimic the available post‐marketing data available for relatively new drugs on the market.

The population consisted of all adults with at least one oral macrolide (azithromycin, erythromycin, clarithromycin) or fluoroquinolone (FQ) (ciprofloxacin, ofloxacin, levofloxacin, moxifloxacin) prescription during the observation period. Participants were eligible for inclusion at the latest of 1 year post‐registration or their 18th birthday. Two active comparators (amoxicillin and cefalexin) were chosen from different antibiotic classes but with similar indications to macrolides and FQs.

The risk window lasted 30 days in the primary analysis and began on the day after the prescription date, to avoid diagnoses made during a medical visit on the same day as the prescription that are unlikely to be related to the drug of interest (DOI). The day of prescription was separated out from all other observation time. Given the length of the observation period and the self‐controlled nature of the design, we conducted a crude primary analysis that is, we assumed that there were no relevant time‐varying confounders. More details on how we handled multiple prescriptions and overlaps can be found in the Supporting Information.

### 2.5 Statistical Analyses and Measures of Performance

We used a conditional Poisson regression to estimate incidence rate ratios (IRRs) and incorporated active comparators using both the simple ratio, and effect modifier models. In the simple ratio model, the effect for the DOI and for the comparator are estimated in two separate case series. The measure of interest is then the ratio of the two IRRs obtained. Ninety‐five percent confidence intervals (CIs) were calculated as CI = exponential of (ln(IRR) ± 1.96 × total standard error) [21]. In the nested model, the total effect was obtained in a single regression with nested exposure variables, which is described in detail in Schultze et al. [10].

Diagnostic performance was measured using sensitivity and specificity with respect to the UK product labels and computing the resulting area under the curve (AUC) of the receiver operating characteristics (ROC). A common reason for the inability to highlight safety signals is insufficient statistical power to highlight safety outcomes, which are often rare. We therefore also looked at the performance of the drug‐outcome pairs where there was apparent sufficient power. We considered satisfactory power for drug‐outcome pairs with at least five events in the risk window, and where the upper bound of the 95% confidence of the IRR was < 2 for outcomes with no evidence for an association; for example, IRR = 1.55 (95% CI: 0.90–2.10) would not be considered satisfactory power.

### 2.6 Sensitivity Analyses

While the main analysis only incorporated the first ever recorded event as an outcome definition to ensure they were incident cases, we explored an all‐events analysis with a 12‐month event‐free period to capture possible repeated incident cases. All included events had to occur within the follow‐up period of the patient with at least a 12‐month difference from the previous occurrence of the same outcome.

### 2.7 Timeliness of Detection

An important aspect of the performance of methods for signal detection lies in their ability to detect ADRs early. To determine whether SCCS could have highlighted positive controls before they were added to labels, we used summary of product characteristics (SPCs) from 1997 to 1998 for the same formulations for both FQs and macrolides as the ones used to create the reference set. We explored whether positive controls highlighted with sufficient power during the 1997/1998 observation period were already present on labels at the time.

## 3 Results

### 3.1 Description of the Study Population

The mean number of antibiotic prescriptions during the observation period varied from 2.7 to 5.7 across different drug use populations (Table 1). The mean age was 60.3 years old at the start of follow‐up. More women than men were included in the study, and the proportion varied substantially by drug. The mean length of follow‐up was 684.7 days (median 729). 7.4%–11.5% of the patients died during the observation period with some slight variation across exposed groups. Power was insufficient for all outcomes with moxifloxacin, and sufficient only for four and two outcomes with levofloxacin and ofloxacin, respectively.

**TABLE 1:** Key characteristics of the population by drug class in the 2017/2018 observation period.

	FQs	Macrolides	Amoxicillin	Cefalexin
Number of patients	34 094	123 290	261 853	22 652
Age at cohort entry				
Mean (SD)	67.2 (17.3)	63.7 (18.8)	64.6 (18.5)	69.8 (18.6)
Median (IQR)	71 (24)	67 (28)	68 (27)	75 (24)
Sex (%)				
Female	53.8	62.7	58.6	75.7
Male	46.2	37.3	41.4	24.3
Mean length of follow‐up (days)	686.3	689.0	689.4	678.8
Number of antibiotic prescriptions*				
Mean (SD)	5.1 (8.0)	3.8 (6.2)	2.7 (4.3)	5.7 (9.0)
Median (IQR)	3 (4)	2 (3)	2 (2)	3 (4)
Proportion of people dying during the observation period (%)	9.2	7.4	7.8	11.5

* of antbiotics considered in this study.

### 3.2 Main Analysis

The overall sensitivity and specificity of the SCCS without an active comparator in the 2017/2018 observation period were 0.31 and 0.77, respectively in CPRD Aurum for all drug‐outcome pairs with at least one event during the risk period (Table 2). Sensitivity increased when restricted to drug‐outcome pairs with satisfactory power, which represented less than 50% of the initial reference set. The sensitivity was 0.57 whilst the specificity was unchanged at 0.77. Specificity increased up to 0.89 with amoxicillin as an active comparator; however sensitivity decreased to 0.18. Cefalexin achieved a higher sensitivity (0.29) than amoxicillin as an active comparator, but a slightly lower specificity (0.87).

**TABLE 2:** Measures of performance for the SCCS in CPRD—2017/2018 observation period—simple ratio model.

	All pairs with at least one event during the risk and baseline periods^a^			Pairs with enough power		
	No comparator	Amoxicillin	Cefalexin	No comparator	Amoxicillin	Cefalexin
Number of pairs	129	129	129	72	72	64
Sensitivity	0.31	0.10	0.16	0.57	0.18	0.29
Specificity	0.77	0.94	0.94	0.77	0.89	0.87
PPV	0.69	0.73	0.81	0.83	0.73	0.80
NPV	0.40	0.38	0.40	0.47	0.41	0.41
AUC	0.54	0.52	0.55	0.67	0.54	0.58

^a^ Thirty three drug outcome pairs not looked at because no events.

### 3.3 Nested Model

The nested and simple ratio models led to similar results in terms of IRRs when using active comparators.

### 3.4 Sensitivity Analysis: All Events With a One‐Year Outcome‐Free Period

Although there were more pairs with satisfactory power than in the primary analysis, performance was slightly decreased to a 0.50 sensitivity and 0.61 specificity in the no comparator analysis (Supporting Information). Sensitivity and specificity were similar to the main analysis when using an active comparator.

### 3.5 Comparison Between Analytical Approaches With and Without Active Comparators

**FIGURE 2:**
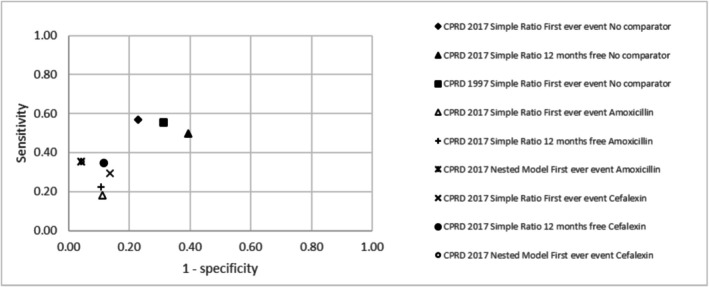
Performance plot for different analytical approaches with and without active comparators.

Two clusters of points appeared when plotting the sensitivity against one minus the specificity for the different analytical approaches investigated in this study, for drug‐outcome pairs with sufficient power (Figure 2). The no‐comparator group had a consistently higher sensitivity compared to the comparator group, but a lower specificity.

### 3.6 1997/1998 Observation Period

Statistical power was low for the 1997/1998 observation period. There were no outcomes with sufficient power for moxifloxacin and levofloxacin, and very few for ofloxacin and erythromycin. Power was acceptable for most outcomes with clarithromycin and ciprofloxacin. In total, 43 drug‐outcome pairs were powered enough (26.5% of the reference set). Power was too low to compute results using active comparators.

Sensitivity was 0.56 and specificity was 0.69 in the 1997/1998 observation period without active comparators for the drug‐outcome pairs which had enough power. Out of the 24 positive controls identified with enough power in the 2017/2018 period, 9 (37.5%) were false negatives in 1997/1998. Power was lacking as per our criteria for eight of them in 1997/1998.

### 3.7 Timeliness of Detection

Out of the 14 positive controls highlighted with enough power during the 1997/1998 observation period, 12 were also identified in 2017/2018 (Table 3). According to information present on the SPCs from 1997 to 1998, 5 of the 14 outcomes highlighted in 1997/1998 were not present on labels in 1998 and added since then, and 9 outcomes were already labelled at the time.

**TABLE 3:** List of positive controls (i.e., labelled in 2018) identified as signals of disproportionality in 1997/1998, giving label status in 1997/1998 and whether it was also highlighted as a signal of disproportionality in 2017/2018.

Drug	Outcome	Present on label in 1997/1998	Highlighted 2017/2018
Ciprofloxacin	Hearing loss	Yes	No
Ciprofloxacin	Oedema	Yes	Yes
Ciprofloxacin	Renal failure	Yes	Yes
Ofloxacin	Tendinitis	Yes	Yes
Erythromycin	Hearing loss	Yes	Yes
Erythromycin	Tinnitus	No	Yes
Azithromycin	Hearing loss	Yes	Yes
Azithromycin	Pneumonia	No	No
Clarithromycin	Arrhythmia	Yes	Yes
Clarithromycin	Atrial fibrillation	No	Yes
Clarithromycin	Cellulitis	No	Yes
Clarithromycin	Hearing loss	Yes	Yes
Clarithromycin	Phlebitis	No	Yes
Clarithromycin	Renal failure	Yes	Yes

## 4 Discussion

We assessed the performance of the SCCS with active comparators for signal detection in the UK CPRD using a reference set of appropriate drugs and outcomes. The overall performance of SCCS in CPRD for drug‐outcome pairs with sufficient power achieved a 0.57 sensitivity and 0.77 specificity.

There is no standard definition of what sufficiently good performance is for signal detection, and this depends on how a method is implemented as part of an overall process [22]. In this study, the SCCS performed better than random chance (area under the ROC curve—AUC of 0.5), with an AUC of 0.67. Although the sensitivity was lower than expected for potential reasons explored below, the SCCS identified more than half of the positive controls in CPRD, indicating that it could be a potentially useful method for signal detection using RWD. Power was one of the main limitations in this study: 72/162 (44.4%) drug outcome pairs had satisfactory power in the main analysis, despite a population of 34 094 individuals for FQs and 123 290 for macrolides. Power was particularly low for FQs other than ciprofloxacin. In the sensitivity analysis, including all events with a 1‐year outcome‐free period, measures of performance were slightly decreased compared to the primary analysis: 0.50 sensitivity and 0.61 specificity. This decrease in sensitivity could be explained by a decrease of the effect because there is limited probability that a second event would occur within the risk period. The ideal outcome‐free period length is likely to vary by outcome but this is impractical for signal detection.

### 4.1 Reasons for False Negatives

Since the sensitivity of the method is one of the most important measures of performance for signal detection, we looked for reasons for missed positive controls. The reference set used in this study was solely based on current UK drug labels. While these provide easily accessible and fairly harmonised information on ADRs, they also include variable levels of evidence of a causal association [23]. Moreover, they mainly include broad medical concepts leading to unspecific codelists for outcome ascertainment. Thurin et al. [20] demonstrated a significant variation in the sensitivity and specificity of the SCCS depending on the outcome definition.

### 4.2 Active Comparators

Active comparators enabled at least partial handling of confounding by indication, with an increase in specificity of 10%–30% depending on the comparator and design choices. There were remaining false positives despite using active comparators, particularly for outcomes very prone to time‐varying confounding by indication, for example cellulitis. However, the sensitivity was greatly decreased compared to the no comparator analysis for two reasons. Firstly, when there was no confounding by indication, defined as a null association between an outcome and an active comparator, the width of the CI for the IRR with the DOI was increased by up to 50% if the number of events was low for the comparator. Secondly, a high proportion of outcomes significantly elevated IRR in the primary analysis with active comparators (17/30 for amoxicillin and 8/30 for cefalexin). This could partly reflect time‐varying confounding by indication but may also be an indication that labels do not always represent gold standard information on the absence of a causal association. The minimum power threshold applied in this study may partly hide the decrease in sensitivity due to using an active comparator, but allowed focusing on product‐events where the ability of the active comparator to address the indication bias had more chance to be highlighted.

Although cefalexin led to better sensitivity as an active comparator compared with amoxicillin across the entire dataset, the sensitivity remained very low for both (0.18 for amoxicillin and 0.29 for cefalexin). This argues against the usefulness of active comparators for signal detection, as sensitivity is the primary focus for pharmacovigilance. Alternative approaches could be used to handle confounding by indication, such as a post hoc manual triage of the alerts by indication before any further signal assessment work for example an increased risk of pneumonia following antibiotic consumption is likely due to the indication rather than the drug itself.

### 4.3 Comparison With Other Studies

Previously, the performance of SCCS in a signal detection context varied between 0.57 and 0.74 in terms of the AUC [24]. In these studies, different design choices were applied depending on the outcome to optimise performance. In our study, applying the same design choices to the entire reference set, the AUC was 0.67 in the primary analysis, which is coherent with the studies cited above. Few of the studies having conducted SCCS performance assessment for signal detection reported measures of sensitivity and specificity, making no comparison possible.

Hypothesis testing studies have investigated certain drug‐outcome pairs included in our reference set in CPRD Aurum. For example, an SCCS study conducted in CPRD Aurum between 1997 and 2019 found limited evidence of an increased risk of uveitis following a FQ prescription, using similar design choices as in this study (29 days risk period, 30 days pre‐exposure window) [25]. Compared to non‐use, the RR was 1.11 (95% CI: 0.94–1.30), which was further decreased to 0.95 (95% CI: 0.78–1.16) using cephalosporin as an active comparator. In our signal detection study, the class estimate for FQs and uveitis was slightly higher at 1.26 (0.73–2.20) with a broader 95% CI due to a much shorter observation period. Power was insufficient to generate a precise estimate with cefalexin as an active comparator. Results between these two studies are compatible; however our estimate for this association did not meet our pre‐specified power requirements. Power would have been greater for longer observation periods. Notably, this is not always possible for near‐real‐time post‐marketing signal detection investigating other drug‐outcome pairs.

### 4.4 Timeliness of the Detection

An important aspect of a method's performance for post‐marketing signal detection is to determine whether the method can detect alerts before they are added to labels. However, there is limited information on when adverse events were added to labels and even less on when a signal was first noted. Using the SPCs from 1997 and 1998, 5 of the 14 signals of disproportionate reporting (SDRs) in 1997/1998 were not present on the corresponding drug labels at the time. Therefore, our results demonstrate that SCCS can highlight signals before they were present on labels. However, some drug‐outcome pairs were already labelled in 1998 but did not show evidence of a higher IRR in the 1997/1998 observation period. This was partly due to power but also to other reasons. For example, the association between ciprofloxacin and tendinitis was not highlighted in 1997/1998 although it was already present on labels. Evidence of the association was seen in the 2017/2018 period, indicating a delay in highlighting the signal compared with other sources such as SRs.

In theory, once an ADR is present on a product label, the SCCS assumption that an event does not influence subsequent exposures may be violated, as the clinician could be aware of the association and this may affect prescribing decisions. In practice, this is probably only true for the more important and widely communicated changes. In many cases it is also unlikely that such an event would act as a permanent contraindication.

### 4.5 Strengths and Limitations

This study is the first large‐scale signal detection performance assessment study for a non‐vaccine medication in CPRD. The reference set included 30 outcomes of all organ classes, which is larger than other studies that have investigated methods' performances in a similar way. Although it was limited to antibiotics, the results should generalise to any drug well suited to the SCCS requirements. All drugs and outcomes we selected were theoretically well suited to the SCCS design. This was done using a taxonomy framework incorporating SCCS assumptions, the clinical importance of the outcomes and suitability to the database of interest among other criteria [19]. Such an approach has not yet been widely applied to signal detection and would benefit other studies to optimise performance.

Power was one of the main limitations of this study, despite having used a large database and having included a minimum number of events recorded in CPRD for each outcome at the design stage. Other limitations include adherence to treatment which is not recorded in CPRD and could lead to non‐differential misclassification of exposure which would tend to bias results towards the null. Residual confounding could also impact the performance of SCCS. However, we attempted to reduce confounding at the design stage as SCCS eliminates between‐people confounding, and using an active comparator could reduce time‐varying confounding by indication.

If RWD is useful for signal detection, an important consideration is whether the entire data source should be used. Our results suggest that power is a key challenge for safety signal detection in CPRD with antibiotics. Assuming our results generalise, the sample size we obtained suggests that all of CPRD would need to be used for signal detection if such a capability is deployed, and concerns of reuse and lack of independence for related pharmacoepidemiologic studies would need to be considered carefully [26, 27]. There is the possibility to use different UK EHRs for further signal assessment studies, for example, OpenSAFELY, since it is based on a different set of primary care practices.

Finally, for signal detection in RWD to become widespread, using the SCCS or other methods, more research is needed to better understand the relative strengths and weaknesses of RWD compared to SRs data for drug safety signal detection.

## 5 Conclusions

There is little evidence about safety signal detection using UK primary care EHRs, and our results add to this substantially. Performance of the SCCS for drug safety signal detection in CPRD, when tailoring the choice of the drug outcome pairs to the chosen study design, is in line with but not higher than previous studies that took a less targeted approach. Power was one of the limiting factors despite using commonly prescribed antibiotics. Active comparators, although they addressed confounding by indication, reduced the proportion of positive controls correctly identified.

### 5.1 Plain Language Summary

We explored the performance of a self‐controlled method, where risks of outcomes (potential adverse events) are compared between different time points in each patient, for the detection of ADRs using UK health records. We also explored the added value of a recent methodological development, active comparators, which are drugs used to treat similar health conditions but were not reported to have adverse events of interest and can address some of the remaining bias. We looked at the ability of the method to identify known drug side effects for commonly prescribed antibiotics. The method was able to give a correct result for 57% of positive controls (known adverse drug events) and 77% of negative controls (drugs known not to be associated with certain outcomes), when limited to drug‐outcome pairs with a satisfying number of occurrences. Five drug‐outcome pairs were highlighted by the method before they were present on drug labels, suggesting the method is capable of timely identification of adverse drug events. Overall, the method performed moderately at finding known ADRs in UK health records. Active comparators reduced the number of positive controls identified but improved the correct identification of negative controls.

## Ethics Statement

The study was approved by the London School of Hygiene and Tropical Medicine Research Ethics Committee (Reference: 27650). The UK study protocol was approved by the Independent Scientific Advisory Committee for the Medicines and Healthcare Products Regulatory Agency (No. 22_002362).

## Conflicts of Interest

A.C. is funded by a GSK PhD studentship to undertake this work. A.B. is an employee of GSK and holds stocks and stock options. F.H. is an employee of GSK and holds financial equities in GSK. I.J.D. holds grants and shares from GSK. GSK markets the following drugs: Augmentin. However A.B. and F.H. did not actively participate in the assessment of the labels and choice of outcomes for this methodological study.

## Supporting information


**Data S1:** pds70243‐sup‐0001‐Supinfo.docx.
